# *Braining*, structured physical activity in specialized psychiatry for patients with substance use disorders—a feasibility study

**DOI:** 10.1186/s40814-026-01898-4

**Published:** 2026-08-06

**Authors:** Åsa Anger, Leida Kaaman, Rebecka Mac, Johan Holmberg, Nitya Jayaram-Lindström, Tobias Lundgren, Maria Hagströmer, Carl Johan Sundberg, Lina Martinsson, Sigrid Salomonsson

**Affiliations:** 1 Centre for Psychiatry Research, Department of Clinical Neuroscience, Karolinska Institutet, & Stockholm Health Care Services, Region Stockholm, Stockholm, Sweden; 2 Stockholm Health Care Services, Region Stockholm, Stockholm, Sweden; 3 Division of Physiotherapy, Department of Neurobiology, Care Sciences and Society, Karolinska Institutet, Stockholm, Sweden; 4 Academic Primary Health Care Centre, Region Stockholm, Stockholm, Sweden; 5 Department of Physiology and Pharmacology, Karolinska Institutet, Stockholm, Sweden; 6 Department of Learning, Informatics, Management and Ethics, Karolinska Institutet, Stockholm, Sweden

**Keywords:** Alcohol use disorder, Anxiety, Depression, Exercise, Feasibility, Metabolic disorder, Physical activity, Psychiatry, Substance use disorder

## Abstract

**Background:**

Individuals with substance use disorders (SUD) have poor metabolic and cardiovascular health and a high prevalence of depression and anxiety symptoms. Regular physical activity can improve both somatic and mental health. This study aimed to evaluate the feasibility of an aerobic physical activity intervention (*Braining*) in psychiatric care services specialized in SUD.

**Methods:**

In this uncontrolled open trial with pre-, post-, and 12-month follow-up measures, 22 patients with SUD participated in a 12-week physical activity intervention. Recommended participation was three 30–45-min moderate-to-vigorous interval-based exercise sessions per week combining aerobic and body-weight strength exercises. No minimum session attendance was required for study participation. Feasibility was assessed through recruitment yield, number of exercise sessions attended, and adherence to study procedures. Satisfaction, acceptability, credibility, and negative effects were assessed using self-report questionnaires and adverse event reporting. Physical activity was measured using accelerometry (ActiGraph GT3X) and self-report. Patient-centered outcomes were summarized descriptively.

**Results:**

The mean number of attended exercise sessions was 9 (SD = 7.7). Adherence to ActiGraph measurements was 73% at baseline, 46% mid-intervention, and 32% post-intervention. Completion of self-report assessments was 100% at baseline, 86% post-intervention, and 77% at 12-month follow-up. The mean Client Satisfaction Questionnaire (CSQ-8) score was 27 (SD = 3.9), and no serious adverse events were reported. Descriptive trends suggested reductions in psychiatric symptoms and substance use and improvements in functional status and quality of life, whereas changes in somatic measures were limited.

**Conclusions:**

Acceptability of the intervention was high in the target patient population; however, exercise attendance was low. Participants engaged adequately in study procedures, but adherence to accelerometer-based measurements was low. With further methodological refinements to enhance patient engagement and adaptations to how physical activity is measured in vulnerable patient populations, *Braining* remains a relevant intervention and warrants systematic evaluation in future large-scale controlled trials.

**Study registration:**

The study was registered on 25/12/2021 at ClinicalTrials.gov under ID NCT05186688.

## Key messages regarding feasibility

- Braining had not previously been implemented or evaluated outside the setting in which it was developed. Feasibility of recruitment, adherence to study procedures, exercise attendance, and ActiGraph-based physical activity measurement was uncertain in patients with SUD, a population often underrepresented in controlled research.
- Recruitment and adherence to study procedures were feasible and acceptability was high, but exercise attendance was below the target frequency and ActiGraph-based physical activity measurement was not feasible in its current form.
- Recruitment and study procedures can largely be retained, but refinements are needed to improve engagement and to develop an alternative accelerometer-based measurement strategy before progression to a randomized controlled trial.

## Background

Individuals affected by mental disorders are less physically active than the non-clinical population, suffer from higher rates of poor somatic health, and have shorter life expectancy [1]. Among psychiatric disorders, substance use disorders (SUDs) are a leading cause of disability, constituting a major public health burden and a known factor contributing to mortality [2–4]. Among patients with SUDs, alcohol use disorder (AUD) is most prevalent [5] and represented in high numbers within the addiction care services in Stockholm, where the study was performed. The high prevalence of AUD is clinically important given the well-established association between chronic and episodic heavy alcohol consumption and adverse somatic outcomes, including cardiovascular disease, cancer, dementia, and injury [3]. In the present study we utilize SUD as an umbrella terminology encompassing AUD as well as other substance-related disorders.

Notably, psychiatric comorbidity is common in relation to SUD, often bidirectionally impacting disorder severity and leading to a worsened clinical course and treatment adherence [6–9]. Major depressive disorder and anxiety disorders have a significant and consistent association with SUD [9, 10], in addition to increasing vulnerability to substance abuse [8] and risk of suicidal behavior [8, 9]. There is also high comorbidity with attention deficit hyperactivity disorder (ADHD) [6, 11], with reports of almost 25% of patients with SUD having the diagnosis [12]. In patients with bipolar disorder, previous studies demonstrate a 40 to 70% [13] prevalence rate for AUD and a 25% prevalence for other SUDs [14], with a risk of adverse outcomes such as developing mixed episodes (co-occurrence of manic and depressive symptoms) and suicide [7, 15].

The treatment for SUD includes psychosocial, psychological, and pharmacological approaches, often in combination [4]. In the presence of psychiatric comorbidity, clinical guidelines generally recommend that substance use and co-occurring mental disorders should be addressed simultaneously using an integrated, multidisciplinary approach [16]. Given the complexity of treating co-occurring mental disorders and SUD, there is increasing interest in interventions that can address multiple symptom domains simultaneously. Physical activity has emerged as a promising transdiagnostic strategy that can be integrated into multidisciplinary care or delivered as an adjunctive intervention, with growing evidence of benefits across multiple psychiatric conditions [17–21]. The World Health Organization (WHO) defines physical activity as any bodily movement produced by skeletal muscles that require energy expenditure and has highlighted its importance for health [17]. Exercise is considered a subcategory of physical activity and refers to planned, structured, and repetitive bodily movement performed with the objective of improving or maintaining physical fitness and health [22]. The key health benefits include primary and secondary prevention of cardiometabolic disorders and improvements in mental health, primarily depressive symptoms and anxiety [17–19]. Aerobic physical activity, particularly at moderate to vigorous intensity, is most effective for these benefits [19]. The evidence supporting the positive effects of physical activity on mental health is rapidly expanding [20]. A recent review, encompassing over 120,000 adult participants from specialized psychiatry, somatic care, primary care, and the general population, demonstrated that physical activity has medium-sized effects on depression, anxiety, and psychological distress compared with usual care and should be a cornerstone in the treatment portfolio for these disorders [21].

Physical activity has previously been studied as a potential adjunct treatment for patients with SUD. Moderate-intensity aerobic exercise programs with durations of 12–13 weeks have been most common in this population [23–25]. Mixed results have been reported regarding the beneficial effects related to alcohol and substance use outcomes, namely consumption, abstinence, and relapse. Some studies suggest no effect on consumption rates [25, 26] and that the effect on abstinence is unclear [23]. Others suggest that an exercise intervention could decrease consumption [24]. However, the benefits in terms of improved physical fitness, depressive symptoms, anxiety, and overall quality of life have been more consistent [23–27]. These findings are of vital importance, considering the previously mentioned prevalence of psychiatric comorbidity and metabolic complications in this population. Furthermore, studies conclude that improved quality of life is an important outcome of treatment [23, 27] and that exercise programs could be included in treatment programs to support the recovery process [23].

Although strong evidence demonstrates that physical activity is safe and effective in improving psychiatric and physical outcomes, it remains insufficiently integrated into routine psychiatric care [28]. This is particularly evident within specialized SUD services, where exercise has not yet been systematically implemented as an adjunctive treatment. Existing approaches used in general healthcare, such as Physical Activity on Prescription [29] and Physical Activity Referral Schemes [30], have been insufficiently studied in psychiatric populations, particularly in patients with substance use disorders and comorbid psychiatric conditions [31].

Braining is a clinical innovation consisting of structured exercise and motivational components developed to integrate physical activity into psychiatric care [32]. It originated from a collaborative effort between patients and staff and was subsequently developed into a structured intervention designed to be scalable and implementable within routine services by ordinary multidisciplinary staff. Importantly, its feasibility has not yet been systematically evaluated in patients with SUD, nor outside the clinical setting in which it was originally developed. A feasibility study was therefore considered an important step to examine implementation under new clinical conditions and to inform procedures for a planned randomized controlled trial (RCT).

### Aim

To evaluate the effect of the *Braining* method, an RCT is planned. In preparation for this RCT, the present study was performed to evaluate feasibility of the *Braining* method, evaluate the procedures from the end-user perspective and explore outcome measures relevant for a subsequent trial.

The primary objectives of the study were to assess feasibility via (a) recruitment procedures (eligibility screening, consent, onboarding to the KI platform) and recruitment rate within the predefined period; (b) adherence to *Braining* exercise sessions; (c) adherence to measurements of physical activity, psychiatric symptoms, quality of life, and metabolic variables; and (d) acceptability, credibility, safety, and perceived negative effects of the intervention. The secondary objectives were twofold: first, to evaluate the practical suitability of selected outcome measures for use in a future randomized controlled trial, including feasibility of data collection and their ability to capture descriptive patterns over time; and second, to explore domains of potential relevance for *Braining* as a transdiagnostic adjunctive intervention, including physical activity, substance use, psychiatric symptoms, sleep, quality of life, functioning, and metabolic health.

## Methods

Where applicable, this report follows the Consolidated Standards of Reporting Trials (CONSORT) statement for pilot and feasibility trials [33] and the “Guidelines for reporting nonrandomized pilot and feasibility studies” [34].

### Study design, setting and participants

The study was designed as an uncontrolled open trial, where participants underwent a 12-week exercise intervention—*Braining* [32]. Before and after the intervention and at a 12-month follow-up, the participants were assessed including measurements of physical activity, psychiatric self-assessments, and metabolic health assessments. The study was carried out at two outpatient units within the Stockholm Centre for Dependency Disorders, part of the psychiatric health care services in Region Stockholm, Sweden, during 2022, with a 12-month follow-up conducted during 2023.

The two outpatient units provided specialized care for patients with substance use disorders (SUD), including treatment of common psychiatric comorbidities. The units were located in the same building, with a shared waiting room and reception. However, the units differed in terms of patient population, treatment focus, and clinic size. Unit A was a larger general outpatient addiction clinic (approximately 1500 unique patients in 2022) serving an adult population and providing both pharmacological and psychological treatment, including relapse prevention. In contrast, Unit B was a smaller specialized outpatient addiction clinic (approximately 300 unique patients in 2022) targeting young adults aged 18–25 years, with greater emphasis on psychological treatment and assessment and management of psychiatric comorbidity.

Participants were eligible if they were aged 18 years or older and currently receiving care at either Unit A or Unit B, both specialized psychiatric outpatient clinics for patients with SUD. Psychiatric comorbidity was common in this clinical setting but was not required for study participation.

Exclusion criteria were severe mental disorder, including acute mania or psychosis, or high risk of suicide or violence as determined by clinical staff; medical conditions contraindicating moderate- to vigorous-intensity physical activity, including cardiovascular or pulmonary disease, acute infection, or withdrawal symptoms; physical disability preventing independent participation in the exercise sessions; cognitive or mental impairment precluding participation in group-based exercise; and insufficient proficiency in Swedish.

### The intervention—Braining

*Braining* was developed as a structured clinical intervention for exercise as an adjunct treatment in specialized psychiatry [32]. It comprises a 12-week program including scheduled 30–45-min aerobic group exercise sessions and structured motivational contacts, delivered by a multidisciplinary team (e.g., nurses, psychologists, and physicians) trained in exercise delivery and physical activity counseling in psychiatric care.

Sessions are conducted indoors or outdoors at the clinic and follow a standardized format: (1) warm-up (5–7 min), (2) interval-based aerobic and bodyweight exercises (22–35 min), and (3) cool-down focusing on mobility and balance. The main component combines moderate- to vigorous-intensity aerobic activities (e.g., jogging in place, jumping jacks) with bodyweight strength exercises (e.g., squats, push-ups). The intervention was structured according to the frequency, intensity, time, and type (F.I.T.T.) principle and designed to align with current World Health Organization (WHO) recommendations for physical activity [17].

Sessions are supervised by two staff members with designated roles: an instructor leading the session and demonstrating exercises, and a host providing individual guidance and adaptations. To ensure consistency and safety, staff use a standardized manualized tool, the *Braining* Box, which includes session plans, exercise descriptions, visual materials, and instructional videos developed by an interdisciplinary team. The tool supports delivery at intended intensity levels while allowing individual adaptation [35].

Exercise intensity is guided using the Borg Rating of Perceived Exertion Scale ranging from 6 to 20, with participants instructed to maintain a level of 11–17 [36]. To support implementation of the target intensity during sessions, the host and instructor use verbal cues to help participants achieve and maintain the recommended exertion level.

Furthermore, *Braining* includes a structured motivational support component consisting of scheduled individual counseling visits throughout the 12-week intervention. These include an initial clinical visit with a trained staff member for risk assessment, evaluation of metabolic and psychiatric health, and individualized goal setting related to both physical activity and expected mental and somatic health outcomes. During the intervention, participants attend monthly follow-up visits to review progress, address barriers and facilitators, and receive motivational support informed by principles of motivational interviewing. A final visit is conducted to evaluate goal attainment and overall outcomes.

In addition, each exercise session is preceded by a brief individual check-in with the instructor or host, including assessment of current mental and physical status and short motivational support to facilitate attendance and engagement.

In this setting, four *Braining* exercise session times per week were offered, and the participants were informed of a recommended frequency of three sessions per week, but with no lower limit to participate in the project.

Prior to recruitment, the outpatient units underwent an implementation process for the *Braining* method. The implementation based on the model developed by Fixsen et al. involves several stages: exploration, program installation, initial implementation, and full operation [37]. The exploration stage included assessing prerequisites and needs, anchoring with management and staff, and making the final decision on implementation. Furthermore, a multidisciplinary *Braining* team was created with staff members from both units. The program installation stage involved preparing resources and educating the team about the *Braining* method, structure, and materials. During the initial implementation stage, *Braining* was launched, the structure was put into operation, weekly meetings were held, and *Braining* sessions were scheduled. Finally, the full operation stage involved recruiting patients, having them participate in *Braining* sessions, and completing their 12-week treatment. The research team provided structured support during weekly meetings with the *Braining* team. After the study, the clinic continued with scheduled *Braining* sessions that both previous study participants and other patients could attend.

### Procedure

Recruitment and onboarding procedures were piloted to inform a future randomized controlled trial (registered on clinicaltrials.org under ID NCT06012149). Patients were recruited during a predefined recruitment period, with a maximum planned sample size of 50 participants based on anticipated recruitment capacity and available resources. In line with guidance for feasibility studies, the planned sample size was chosen to allow evaluation of the predefined feasibility outcomes, including recruitment procedures, intervention delivery, intervention adherence, and completion of study assessments, rather than statistical power or precision of effect estimates [33, 34].

Clinical staff were instructed to inform patients about the study during regular visits. Additional information regarding the study was available on posters in the waiting rooms. Interested patients received detailed written and oral information about the study at a reoccurring group start-up meeting, where the study and Braining intervention were introduced and participation procedures were initiated as follows: after signing the informed consent form, the participants received a QR code to create an account on a secure online platform provided by the Karolinska Institutet (KI). Upon registration, the participants were automatically assigned a unique study ID. The participants completed a health screening questionnaire, which was developed for this study based on current national and European guidelines regarding health risk assessment and exercise [38, 39]. The health screening questionnaire included body weight, height, age, and current physical activity as well as 12 items covering risk factors for congenital or genetic heart disease, risk factors for cardiovascular events, ongoing infectious disease, chronic respiratory disease and neuromusculoskeletal diseases. Submitted answers were evaluated by the study physician. If the screening revealed potential risk factors for participation in an exercise intervention, the patient provided more detailed responses to the screening questions orally, before the physician decided on eligibility. The patient was excluded and recommended to seek health care if the risk was deemed too high. Patients with a low health risk who fulfilled the inclusion criteria were included in the study, and premeasurements were initiated.

**Fig. 1:**
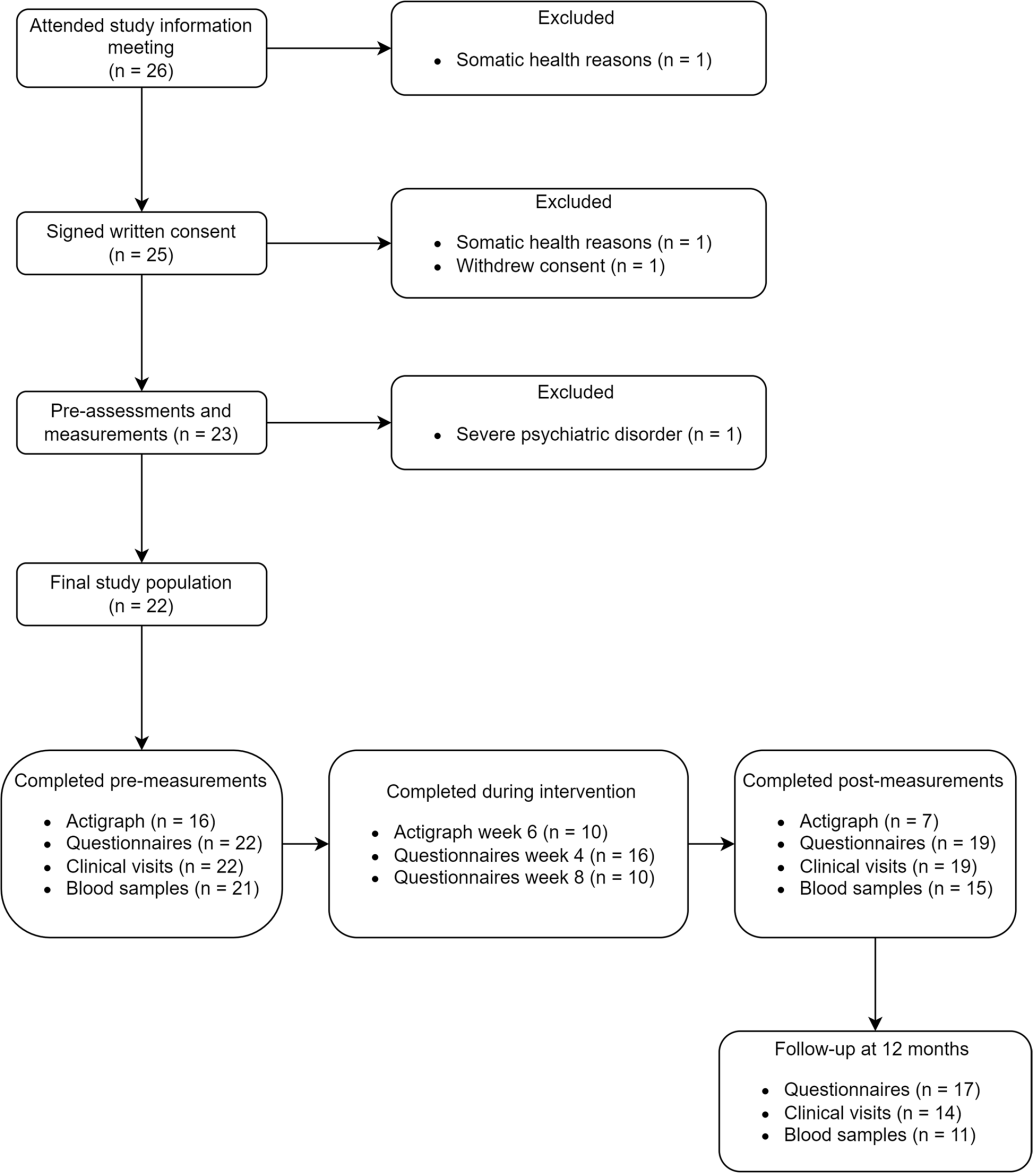
Flow chart. Overview of the participation process, detailing the number of included and excluded patients as well as the completion rates of the study protocol components among the final study population

An overview of the participant flow is presented in Fig. 1. Following inclusion, the *Braining* intervention was initiated with a scheduled clinical visit to trained nurses experienced in motivational interviewing [40]. The participants received the weekly exercise schedule as well as instructions regarding the booking procedure and were asked to set individual goals as an initial motivational strategy.

Physical activity was measured via the ActiGraph GT3x accelerometer (ActiGraph, Pensacola, FL). The accelerometers were initialized by the researchers and handed directly to the participant or via the clinic reception to be picked up during clinical visits. The participants were instructed to wear the accelerometer on the hip [41] during waking hours for seven consecutive days at three time points (pre-, mid- and postintervention). Additionally, participants were instructed to log their daily wear time in a diary used to verify wear time and number of valid days. Another accelerometer measurement was planned at the 12-month follow-up but was canceled because of insufficient adherence at mid- and post-intervention. The outcome measures in the study protocol were amended accordingly.

Self-assessment instruments were administered before, at 4 and 8 weeks, after the intervention and at the 12-month follow-up. All self-assessments were completed on the KI online platform. An automated monthly text message containing a link to the questionnaires was sent to participants via the KI platform. If the participant failed to fill out the forms in time, a series of reminder text messages were generated. Additionally, a research nurse or assistant called the participant on up to 3 occasions.

Physical examinations were performed during the clinical visits at inclusion (see the “ Patient-centered outcomes” section) postintervention and at the 12-month follow-up. Fasting blood samples were obtained either by nurses at the clinic or with a referral to a lab of the participant’s choice at the same time points. Physical examinations at the 12-month follow-up were performed by a research nurse.

Baseline data regarding age and gender were provided at registration on the KI platform. Data regarding current primary and secondary psychiatric diagnoses were extracted from patient medical records and noted at inclusion by an appointed staff member. Medical records were also reviewed post-intervention and at the 12-month follow-up to collect data on Braining session attendance, clinical visits, metabolic measures, and blood samples. Data from all self-assessments were extracted from the KI platform. In parallel, semi-structured qualitative interviews were conducted with a subsample of patients to explore experiences of the intervention and barriers/facilitators for participation. These data will be reported in a separate publication and are not analyzed in the present paper.

### Outcome measurements

#### Feasibility outcomes

Adherence to the physical activity intervention was assessed by the number of *Braining* exercise sessions attended during the 12 weeks intervention period. Based on international physical activity guidelines from the World Health Organization, a target frequency of three moderate-to-vigorous intensity sessions per week was specified [17]. Exercise participation from post-intervention to the 12-month follow-up was also assessed; however, no predefined target was set for this period, as participation after the study period was optional. Adherence to measurements was assessed by group-level completion rates of ActiGraph measurements, questionnaires, clinical visits, and blood samples across time points. Based on the commonly used rule of thumb that missing data exceeding 20% may threaten validity, a completion rate of 80% or higher was considered indicative of feasible adherence to measurements [42].

Acceptability was measured by the Client Satisfaction Questionnaire (CSQ-8), which is designed to rate global satisfaction with treatment. The total score ranges from 8 to 32, where higher values indicate greater satisfaction. The CSQ-8 has high internal consistency and a correlation coefficient of 0.4 with reported changes in symptoms [43]. The cutoff value for adequate treatment satisfaction is commonly set at above 20, with values ranging from 21 to 25 corresponding to an intermediate level and values ranging from 26 to 30 indicating a high satisfaction level [44]. Accordingly, a total CSQ-8 score above 20 was interpreted as indicating adequate acceptability of the intervention.

Expectancy and credibility were measured by the Credibility and Expectancy Questionnaire (CEQ) [45, 46], a scale commonly used in clinical outcome studies. The scale consists of six questions, three questions exploring credibility, and three covering expectancy. For credibility, a mean can be calculated. With respect to expectancy, the question “By the end of the treatment, how much improvement in your symptoms do you think will occur?” is considered representative and was therefore chosen for analysis. The questions are rated on a 0–100% scale with 10-point increments. The questionnaire has high internal consistency between the two factors and adequate test–retest reliability. As no clearly established cutoff values exist for either the credibility subscale or the chosen expectancy question of the CEQ, results were interpreted descriptively rather than used to define progression thresholds.

The Negative Effects Questionnaire (NEQ) [47] evaluates negative effects during psychological treatment. The items explore six validated factors: symptoms, quality, dependency, stigma, hopelessness, and failure. The full instrument as well as individual factors have yielded adequate internal consistency. The questionnaire differentiates between negative effects attributed to treatment and those possibly caused by other circumstances. Given its descriptive nature, NEQ data were used to inform safety monitoring and intervention refinement, rather than to define progression criteria.

Safety was measured as the number and severity of reported adverse events during the exercise intervention period. This information was collected through oral reports during weekly *Braining* staff meetings, where adverse events were one of the standing items.

#### Physical activity outcomes

In clinical studies and settings, accelerometry is considered the “gold standard” for measuring physical activity [48]. In this work, the ActiGraph GT3X accelerometer was used. The ActiGraph accelerations were sampled at 30 Hz and summed over 60 s via ActiLife v.6.13.4 software. The non-wear time was set to > 90 min of consecutive zero counts, allowing for 2 min of nonzero counts [49]. Data were deemed feasible for analysis in measurements with ≥ 4 days and ≥ 10 h per day of valid wear time [50]. Wear time was allocated into steps per day and activity categories in minutes per day based on count-based thresholds: sedentary behavior < 100 counts per min (cpm) [51], low-intensity physical activity 100–1951 cpm and moderate to vigorous intensity physical activity ≥ 1952 cpm [52]. The International Physical Activity Questionnaire (IPAQ) is an internationally used self-assessment instrument for indirect measures of physical activity divided into different levels of intensity and time spent sitting. The short 7-question form was used. The IPAQ has acceptable criterion validity and specificity in the general population, although its sensitivity has been shown to be low [53].

#### Patient-centered outcomes

All self-reported measures were administered digitally and completed by the patients, including assessments of alcohol and drug use, psychiatric symptoms, quality of life and function.

The Alcohol Use Disorders Identification Test Consumption (AUDIT-C) [54] is a 3-item clinical questionnaire that measures alcohol consumption and has high test–retest reliability. The Drug Use Disorder Identification Test (DUDIT) [55] is an 11-item reliable and valid questionnaire covering patterns of current drug use and drug-related problems.

The 9-item Patient Health Questionnaire-9 (PHQ-9) [56, 57] is designed to screen for depression as well as a measure of depression severity. Its sensitivity for detecting major depressive disorder has been thoroughly tested, and it has yielded good results. The 7-item Generalized Anxiety Disorder Questionnaire (GAD-7) [58] is one of the most frequently used measures for general anxiety because of its validity and diagnostic reliability as well as sensitivity to change during treatment. The Insomnia Severity Index (ISI) [59] is a reliable and valid self-rating questionnaire for evaluating perceived sleep difficulties. It is sensitive to treatment response and has satisfactory internal consistency.

The Brunnsviken Brief Quality of Life Scale (BBQ) [60] was developed to measure self-rated subjective quality of life and covers six identified life areas, such as leisure, learning and self-view. It has been tested and validated in both nonclinical and clinical subjects, including psychiatric populations. Moreover, it has shown adequate reliability and consistency. The World Health Organization Disability Assessment Schedule 2.0 (WHODAS-2) [61] measures health and disability. The brief version used in this study consists of 12 items evaluating function over six life domains, including cognition, mobility, self-care, getting along, life activities and participation. The WHODAS-2 has shown high validity and reliability. The EQ5D-5 L [62] is a well-known, reliable, and valid instrument for measuring health-related quality of life. It consists of five dimensions—mobility, self-care, usual activities, pain/discomfort and anxiety/depression—and a visual analog scale for overall health ranging from 0 to 100. The Treatment Inventory of Costs in Patients with Psychiatric Disorders (TiC-P) [63] covers healthcare consumption and production losses in patients with mental disorders. It consists of two parts that can be used separately. In this study, the second part, measuring production losses through absence from and reduced efficiency of work, was administered. It has been regarded as feasible and reliable, with adequate consistency.

Somatic health was evaluated via physical examination and metabolic blood markers. During physical examination at clinical visits, blood pressure (BP), heart rate, waist circumference (WC) and body mass index (BMI) were measured. To minimize interindividual variability, the measurements were performed in accordance with a predetermined protocol. Blood pressure was measured while the participants were in a seated position with either an automatic or manual blood pressure cuff. WC was measured according to the WHO WC-mid [64].

To evaluate the metabolic profile of the participants, levels of blood lipids (triglycerides, low-density lipoprotein, high-density lipoprotein), blood glucose and HbA1c were measured.

### Statistical analysis

In line with the feasibility design, analyses were primarily descriptive. Exploratory analyses of changes over time in patient-centered outcomes were conducted to inform outcome selection for a future randomized controlled trial. All continuous variables are presented as mean with standard deviation (SD) or median with range. Categorical variables are presented as frequencies and proportion (percentages). Adherence to the intervention and study procedures (exercise participation, ActiGraph measurements, questionnaires, clinical visits, and blood samples) was summarized descriptively. Acceptability, credibility, and negative effects (CSQ-8, CEQ, and NEQ) were analyzed descriptively. Missing data were not imputed, and analyses were based on available data.

## Results

### Recruitment feasibility

During the predefined recruitment period January to April 2022, start-up meetings were held on seven occasions during March and April. In total, 26 patients attended start-up meetings. During recruitment, two participants were excluded due to somatic health risks, and one withdrew consent, resulting in a total sample of 23 participants. During pre-assessment, one additional participant was excluded after developing an acute episode of severe mental disorder, yielding a final study population of 22. The participant flowchart is presented in Fig. 1. At the 12-month follow-up, 7 participants had ongoing medical care contact at clinics A or B.

Table 1 shows the baseline characteristics of the final study sample. Registered diagnoses extracted from medical records showed that 20 of 22 participants had at least one registered SUD diagnosis, of which AUD was the most common (*n* = 13). The two remaining participants did not have a registered SUD diagnosis in the medical records but were receiving ongoing treatment for substance use problems at the participating clinics. With respect to other psychiatric diagnoses, hyperkinetic disorders were the most common (*n* = 5), followed by anxiety disorders (*n* = 3).

**Table 1:** Baseline characteristics of the study population

Demographics	Participants (*N* = 22)
Age, mean (SD)	38.7 (12.3)
Male, *n* (%)	18 (81.8)
Employment status and work absence rate	Participants (*N* = 19)
Currently employed (%)	8 (42.1)
Sickness absence last 4 weeks, *n* (%)	6 (31.6)
Diagnoses	Participants (*N* = 22)
Alcohol use disorders (F101, F102), *n*	13
Cannabis use disorder (F121), *n*	2
Stimulant use disorder (F152), *n*	1
Polydrug use disorders (F191, F192), *n*	5
Hyperkinetic disorders (F900, F900B, F900C), *n*	5
Bipolar disorders (F319), *n*	1
Depressive disorders F321), *n*	1
Anxiety disorders (F401, F412), *n*	3
No specified diagnosis (Z032), *n*	1
Singular diagnosis, *n*	11
Baseline physical activity	Participants (*N* = 16)
Sedentary time, minutes mean (SD)	504 (127)
Moderate physical activity, minutes mean (SD)	52.1 (42.5)
Vigorous physical activity, minutes mean (SD) Steps per day, mean (SD)	2.4 (8.0) 9510 (4870)

Demographic data and employment, diagnostic groups and baseline physical activity. Note: Diagnostic groups are classified according to the 2019 version of the International Classification of Diseases (ICD-10). Diagnoses were extracted from medical records and are displayed in a summarized manner, meaning that a participant can contribute to more than one diagnosis. Baseline physical activity is measured with ActiGraph and presented as minutes or steps per valid day

### Adherence to intervention and measurements

The exercise participation rate during the 12-week intervention period was a mean of 9 and a median of 8 *Braining* exercise sessions (SD = 7.7, range = 1–30). Eighteen participants attended at least 3 sessions. The two most active participants attended 29 and 30 sessions, respectively. At the 12-month follow-up, 27% (*n* = 6) continued to participate in *Braining* sessions after the post-intervention period ended. For these participants, the number of sessions attended ranged from 2 to 90, with a mean of 20.7 (SD = 34.7) and a median of 4 sessions.

Adherence to the study protocol assessment components included ActiGraph measurements, questionnaires, clinical visits, and blood samples and are reported in Fig. 1. Adherence to ActiGraph measurements varied and diminished with time, with 16 (72.7%) valid measurements at baseline, 10 (45.5%) at week 6, and 7 (31.8%) at week 12. Out of the 16 valid baseline measurements, 2 contained 4 valid days, 1 had 5 valid days, 5 had 6 valid days and 8 measurements had 7 valid days. On valid days, the mean wear time was 802.6 (SD = 130.5) min.

The rate of completed questionnaires varied but was generally high, with 22 (100%) at baseline, 19 (86%) after treatment and 17 (77.3%) at the 12-month follow-up. The rate was the lowest at week 8 (45.5%, *n* = 10).

The rates of completed clinical visits and blood samples were generally high at baseline and declined over time: 22 (100%) to 14 (63.6%) and 21 (95.5%) to 11 (50%), respectively.

### Feasibility ratings

The CSQ-8 was completed by 17 participants, and the mean value was 26.8 (SD = 3.9), out of 32. This corresponds with response 3, mostly satisfied, or 4, very satisfied, for each item. Item 3, “did the treatment meet your need,” received the lowest mean score of 2.99 (SD = 0.66), whereas item 4, “would you recommend the treatment to a friend,” received the highest mean score of 3.71 (SD = 0.47). Among the 22 participants, 21 completed the CEQ. The mean value of the first 3 items representing treatment credibility was 7.3 (SD = 1.18), out of 9. The mean value of expected improvement in symptoms was 51.9% (SD = 21.2).

The NEQ was completed by 19 participants. Overall, reported negative effects were few and low. All the scores for negative effects were 1 (out of 4). The most reported items were as follows: increased sense of stress (*n* = 4, 21.1%), resurfacing of unpleasant memories (*n* = 3, 15.8%), thinking that the problem they were seeking help for could not improve (*n* = 3, 15.8%), not always understanding the treatment (*n* = 3, 15.8%), feeling that the treatment did not suit them (*n* = 3, 15.8%) and thinking that they developed a dependency on the treatment (*n* = 2, 10.5%). The reported negative effects were not reported to affect the participants in a negative way.

During the 12-week study period, there were no reports of any serious adverse events. One minor adverse event, a migraine episode, was reported.

### Physical activity outcomes

Owing to low ActiGraph adherence at weeks 6 and 12, no meaningful analysis of changes could be conducted.

Due to a technical error in the digital administration of the IPAQ, which made it unclear whether the responses referred to minutes per day or per week, the data were considered unusable.

### Patient-centered outcomes

The measures of psychiatric self-assessments are presented in Table 2. With respect to the psychiatric symptom ratings, the PHQ-9 score was above the clinical cutoff of 10 for depression [65] in 64% of the participants before treatment, 37% after treatment, and 29% at the 12-month follow-up. The GAD-7 score was above the clinical cutoff of 8 for generalized anxiety [65] in 40% of participants before treatment, 21% after treatment, and 18% at the 12-month follow-up. The ISI was above the clinical cutoff of 11 for detecting insomnia [59]: 55% before treatment started, 32% after treatment and 28% at the 12-month follow-up.

**Table 2:** Measures of psychiatric symptoms including substance use, functional status, and quality of life

Measure	Pre M(SD)	4 weeks M(SD)	8 weeks M(SD)	Post M(SD)	Follow-up M(SD)
AUDIT-C	3.9 (4.4)	2.2 (3.8)	2.0 (3.1)	1.8 (2.7)	2.4 (3.3)
DUDIT	12.6 (15.1)	12.1 (14.3)	7.8 (10.5)	6.3 (8.5)	3.1 (5.8)
PHQ-9	11.55 (5.83)	10.00 (4.79)	10.20 (6.41)	7.79 (5.42)	6.44 (4.41)
GAD-7	7.62 (3.97)	8.12 (4.62)	7.5 (5.72)	6.0 (4.7)	4.7 (3.4)
ISI	12.5 (6.3)	11.5 (5.3)	10.9 (6.9)	8.5 (6.0)	8.9 (5.7)
BBQ	37.5 (19.7)	47.7 (23.8)	48.7 (27.6)	51.0 (18.4)	50.9 (27.8)
WHODAS-2	29.5 (21.7)	21.5 (11.8)	22.5 (14.9)	21.1 (15.5)	41.7 (43.2)
EQ5D-5 L VAS	58.2 (17.1)	62.5 (14.0)	59.3 (19.3)	62.9 (16.7)	69.4 (20.82)
EQ5D-5 L Index	0.894 (0.062)	0.909 (0.050)	0.896 (0.063)	0.916 (0.061)	0.917 (0.053)

Means (M) and standard deviations (SD) for self-assessed measurements of substance use, mental health, and quality of life preintervention, 4 and 8 weeks in, post- and 12 months after intervention. *Note.* *AUDIT-C* Alcohol Use Disorders Identification Test for Consumption, *DUDIT* Drug Use Disorders Identification Test, *PHQ-9* Patient Health Questionnaire-9, *GAD-7* General Anxiety Disorder 7-item scale, *ISI* Insomnia Severity Index, *BBQ* Brunnsviken Brief Quality of Life Scale, *WHODAS-2* World Health Organization Disability Assessment Schedule 2.0, *EQ-5D* EuroQol-5 dimension, *VAS* visual analog scale

The measures of metabolic health pre- and post-intervention and at 12 months follow-up are shown in Table 3. Overall, the values indicate adequate metabolic health, although mean BMI and mean waist circumference were slightly above normal range [66, 67].

**Table 3:** Measures of metabolic outcomes

Measure	Pre M(SD)	Post M(SD)	Follow-up M(SD)
Blood pressure Systolic	131 (13)	128 (12)	124 (9)
Blood pressure Diastolic	84 (11)	79 (12)	81 (8)
Pulse	76 (15)	75 (11)	75 (12)
Waist circumference	98.4 (15.6)	94.1 (17.5)	98.1 (15.4)
BMI	26.5 (5.9)	26.4 (6.15)	27.2 (5.47)
Total cholesterol	5.0 (1.1)	5.1 (1.0)	5.1 (1.4)
LDL	3.2 (0.9)	3.2 (0.8)	3.2 (1.2)
HDL	1.3 (0.3)	1.3 (0.3)	1.4 (0.3)
LDL/HDL quota	2.5 (0.8)	2.5 (0.7)	2.4 (0.9)
Triglycerides	1.2 (0.7)	1.4 (0.8)	1.1 (0.6)
Blood glucose	5.5 (0.6)	5.5 (0.6)	5.6 (0.3)
HbA1c	34 (3)	35 (4)	35 (3)

Means (M) and standard deviations (SD) for metabolic blood tests pre, post, and 12 months after intervention

*BMI* body mass index, *LDL* low-density lipoprotein, *HDL* high-density lipoprotein

## Discussion

This study examined the feasibility of delivering the *Braining* exercise intervention, in routine psychiatric care, using patients with SUD as the first clinical test group for a planned transdiagnostic randomized controlled trial. The results on the feasibility outcomes were mixed. Recruitment goals were met; however, attendance at the voluntary *Braining* exercise sessions was below the target frequency, indicating a need for further refinement. Questionnaire and metabolic assessment adherence were acceptable, whereas adherence to ActiGraph-based physical activity monitoring was low and deemed not feasible in its current form. Participants reported high satisfaction and credibility and few negative effects, along with no reported adverse events, supporting the intervention’s acceptability and safety in this patient group. Patient-reported outcomes varied over time; however, these descriptive findings cannot be attributed to the intervention and primarily served to inform the feasibility and responsiveness of the selected measures for the future trial.

The achieved sample allowed evaluation of the predefined feasibility outcomes and provided valuable information regarding recruitment procedures, intervention delivery, and study assessment procedures to inform the subsequent randomized controlled trial.

A target frequency of three *Braining* exercise sessions per week was specified at the study level; however, participation was voluntary and no predefined attendance level was required. At the group level, attendance remained below the intended target, with participants attending on average fewer than one session per week and showing substantial interindividual variation. Similar participation levels have been reported in exercise interventions targeting comparable clinical populations [68–70], as well as in our evaluation of clinical data from the first years of implementation of *Braining* in regular care [32].

Given that even modest increases in moderate- to vigorous-intensity physical activity may yield health benefits in this population [17], continued evaluation of the intervention remains relevant. This is further supported by a recent review reporting that as little as one exercise session per week is associated with statistically significant improvements in anxiety and depressive symptoms [71]. Importantly, the observed attendance levels should not be interpreted as an argument for lowering adherence expectations. Rather, they highlight the need to better understand barriers and facilitators of participation and to use this knowledge to refine the intervention. The wide variation in attendance further suggests the presence of subgroups with differing engagement patterns, which should be further explored in future intervention development. Finally, participation in *Braining* exercise sessions represented only one component of overall physical activity, and participants were not restricted from engaging in additional exercise outside the clinic. Accordingly, session attendance should not be interpreted as a proxy for total physical activity exposure during the intervention period.

Adherence to accelerometry was suboptimal at baseline (73%) and declined at subsequent time points (46–32%). Compliance with ActiGraph diaries was poor, limiting their use in data processing and contributing to a high proportion of invalid measurements. Similar challenges have been reported previously; for example, in a Swedish study using ActiGraph devices in individuals with alcohol use disorder, only 42% of participants provided usable device-measured physical activity data [72].

Despite these measurement challenges, baseline ActiGraph data were available for analysis and indicated relatively high mean levels of moderate-intensity physical activity compared with other psychiatric populations [73–75]. Mean steps per day were also higher than expected and comparable to levels reported in healthy populations [76]. These unexpectedly high activity levels may partly reflect measurement reactivity. Selection bias may also have contributed, as participants who were more motivated or physically active may have been more likely to provide valid measurements. Interestingly, baseline activity was similar to that reported in the Swedish AUD study [72].

Completion of questionnaires and clinical visits was high at baseline (100%) and post-intervention (86%), supporting feasibility of these procedures. In contrast, adherence to blood sampling was lower post-intervention (95% pre-intervention; 68% post-intervention), which may be explained by the need to attend an external laboratory. Questionnaire completion at weeks 4 and 8 was lower (73–45%), suggesting that reducing questionnaire burden and optimizing measure selection may improve adherence in this population. At the 12-month follow-up, participation declined compared with post-intervention (clinical visits 64%; blood samples 50%), which was expected given that only one-third of participants remained registered at the clinics and available for reminders. This reflects routine SUD care, where treatment episodes are often shorter than in general psychiatric outpatient care and patients more frequently discontinue contact with services. Questionnaire completion remained relatively high (77%), indicating that digital administration via the KI platform was feasible and acceptable.

Evaluating treatment credibility, satisfaction, and potential negative effects was central to this study. At inclusion, participants rated the intervention as credible, with scores comparable to those reported in similar studies [77, 78]. Expectations of improvement were relatively modest; the mean expectancy of 52% was lower than that reported in a comparable study of transdiagnostic cognitive behavioral therapy (the Unified Protocol) [45]. This difference is not unexpected, given that CBT is an established and guideline-recommended first-line treatment for common mental disorders such as depression and anxiety [65, 79, 80].

Satisfaction with the intervention at post-intervention was high, with participants rating CSQ-8 items at an average of 3 out of 4, indicating that participants perceived the intervention as being of good quality and meeting most of their needs. Self-reported negative effects were rare; however, the NEQ has not been validated for use in physical activity interventions. Overall, high satisfaction and low levels of negative effects support acceptability in this clinically complex population. However, the discrepancy between participant satisfaction and exercise attendance suggests that further intervention refinement and evaluation are needed before proceeding to a larger transdiagnostic randomized controlled trial.

No serious adverse events were reported, indicating an acceptable level of safety for both the exercise intervention and the recruitment process. This supports continued use of the medical screening procedure in future studies. Minor adverse events were rare but may have been underreported, as previous exercise therapy research has reported higher rates (e.g., a 19% increase in minor adverse events) [81].

Overall, the patient-reported outcome measures were feasible to administer in this population and were able to capture variation over time across several symptom and functioning domains, supporting their suitability for inclusion in a future randomized controlled trial. Patterns observed in self-reported substance use and psychiatric symptoms likely reflect multiple concurrent influences, including ongoing treatment, and should therefore not be interpreted as intervention effects. Levels of quality of life and functional impairment were consistent with previous clinical applications of the BBQ, WHODAS 2.0 (12-item), and EQ-5D-5L, further supporting the relevance of the selected measures [60–62]. In contrast, the somatic outcome measures appeared less informative over the study period. Baseline somatic assessments indicated a high prevalence of abdominal obesity [66], but most metabolic blood markers remained within the normal range at all time points. In order to capture potential metabolic health effects, individuals with greater metabolic burden may be particularly important to target or stratify in future trials.

### Limitations

This pragmatic feasibility study was conducted in routine clinical care and focused on patients with SUD, a clinically complex population that is often underrepresented in controlled research [24]. The design, which included a long-term follow-up, allowed us to pilot and evaluate multiple aspects of the *Braining* intervention and to generate information relevant for the design of a future randomized controlled trial.

However, several limitations should be acknowledged. First, progression criteria were not predefined. While this may limit the strength of feasibility conclusions, the primary aim of the study was to generate empirical information on recruitment procedures, intervention delivery, adherence to assessments, and acceptability to inform the specification of progression criteria in a subsequent randomized controlled trial. Second, the open trial design did not allow testing of procedures related to randomization or control conditions. This was expected given the exploratory nature of the feasibility design. Third, data on the recruitment process prior to the start-up meetings were not systematically collected. As a result, the total number of patients approached and reasons for declining participation are unknown. This limited the ability to evaluate recruitment reach and potential differences in recruitment between Unit A and Unit B, highlighting the importance of prospectively documenting the full recruitment process in future trials. Fourth, the intervention included a flexible, non-mandatory recommendation regarding exercise frequency, which resulted in substantial interindividual variation in participation. While this pragmatic approach reflects routine clinical care and was intentional, it limits conclusions regarding adherence under more standardized conditions. Fifth, psychiatric diagnoses were extracted from medical records and were not verified through structured diagnostic interviews, which resulted in an incomplete list of diagnoses. This approach reflects routine clinical practice but limits diagnostic precision. Future studies should include structured diagnostic assessments to enable more detailed characterization of the study population. Finally, the broad inclusion strategy resulted in a heterogeneous sample regarding treatment stage, physical health, symptom severity, baseline physical activity and psychiatric diagnoses. While this reflects routine clinical populations, it may have complicated the interpretation of feasibility outcomes such as recruitment, attendance, adherence, and participant acceptability across subgroups. In particular, no upper threshold for baseline physical activity was applied, resulting in inclusion of participants with relatively high activity levels. This finding has informed refinements to the design of the planned randomized controlled trial.

### Clinical implications and future studies

Given the feasibility design, the primary implications relate to utilizing the current knowledge gained to further refine the intervention and study procedures prior to the planned randomized trial. Overall, integration of the exercise intervention into clinical care, recruitment procedures, and data collection appeared feasible. However, substantial variability in exercise participation suggests that additional work is needed to determine which aspects of the *Braining* method may require modification to better support session attendance. To further explore barriers and facilitators of participation, participants’ experiences will be examined through qualitative interviews conducted alongside the intervention. This will include motivational, practical, and contextual factors.

The ActiGraph protocol, including the diary and on/off routine, may have been too demanding for this population. In future studies, a smaller accelerometer enabling 24-h wear without diary requirements could be considered to improve feasibility. To reduce the risk of losing information on overall physical activity, brief and easily accessible self-report measures may be collected regularly as a complement to accelerometers. The Swedish National Board of Health and Welfare’s physical activity indicator questions (Socialstyrelsen) could represent a suitable option [82]. Additionally, future studies may incorporate objective measures, such as heart rate monitors, to further evaluate exercise intensity and optimize intervention delivery.

Future studies may also benefit from enriching the sample with individuals with low baseline physical activity and elevated metabolic risk, which could increase the likelihood of detecting changes in metabolic outcomes. Finally, the pre-inclusion health screening procedure appeared valuable and may remain important for safe integration of physical activity in psychiatric care and in the forthcoming trial.

## Conclusions

This feasibility study suggests that *Braining* can be delivered as an adjunct exercise intervention in specialized psychiatric services for patients with SUD. Participants reported high acceptability, whereas *Braining* exercise session attendance was below target frequency. Accelerometer-based measurement was not feasible in its current form. With methodological refinements to support user engagement and improve physical activity measurement, *Braining* warrants systematic evaluation in future large-scale controlled trials.

## Data Availability

The datasets generated and analyzed during the current study are not publicly available owing to individual privacy even though they are pseudonymized but are available from the corresponding author upon reasonable request. Data are located in controlled access data storage at KI.

## Abbreviations

ADHD: Attention deficit hyperactivity disorder
AUD: Alcohol use disorder
AUDIT-C: Alcohol Use Disorders Identification Test Consumption
BBQ: Brunnsviken Brief Quality of life Scale
BMI: Body mass index
BP: Blood pressure
CEQ: Credibility and Expectancy Questionnaire
Cpm: Counts per minute
CSQ-8: 8-Item Client Satisfaction Questionnaire
DUDIT: Drug Use Disorders Identification Test
EQ5D-5 L: The EQ5D™ is a trademark of the EuroQol Group and a self-assessment instrument for describing and valuing health status
GAD-7: 7-Item Generalized Anxiety Disorder Questionnaire
IPAQ: International Physical Activity Questionnaire
ISI: Insomnia Severity Index
KI: Karolinska Institutet
NEQ: Negative Effects Questionnaire
PHQ-9: 9-Item Patient Health Questionnaire
RCT: Randomized controlled trial
SUD: Substance use disorder
WC: Waist circumference
WHO: World Health Organization
WHODAS-2: The World Health Organization Disability Assessment Schedule 2.0
